# Use of laboratory-developed assays in global HIV-1 treatment-monitoring and research

**DOI:** 10.1038/s41598-023-31103-y

**Published:** 2023-03-20

**Authors:** Jemima Malisa, Mark Manak, Clive Michelo, Nesrina Imami, Catherine N. Kibirige

**Affiliations:** 1 IAVI, Human Immunology Laboratory, Imperial College London, Chelsea and Westminster NHS Foundation Trust, 369 Fulham Road, London, SW10 9NH UK; 2 Turesol Consulting, King of Prussia, PA USA; 3 Zambia Emory HIV Research Project, Lusaka, Zambia; 4 Centre for Immunology and Vaccinology, Imperial College London, Chelsea and Westminster NHS Foundation Trust, 369 Fulham Road, London, SW10 9NH UK

**Keywords:** Health care, Diagnosis, Laboratory techniques and procedures, Biomarkers, Prognostic markers, Molecular medicine

## Abstract

There has been a surge in the emergence of HIV-1 drug resistance in Low and Middle-Income Countries (LMICs) due to poor drug-adherence and limited access to viral load testing, the current standard for treatment-monitoring. It is estimated that only 75% of people living with HIV (PLWH) worldwide have access to viral load testing. In LMICs, this figure is below 50%. In a recent WHO survey in mostly LMICs, 21 out of 30 countries surveyed found HIV-1 first-line pre-treatment drug resistance in over 10% of study participants. In the worst-affected regions, up to 68% of infants born to HIV-1 positive mothers were found to harbour first-line HIV-1 treatment resistance. This is a huge public health concern. Greater access to treatment-monitoring is required in LMICs if the UNAIDS “third 95” targets are to be achieved by 2030. Here, we review the current challenges of viral load testing and present the case for greater utilization of Laboratory-based assays that quantify intracellular HIV-1 RNA and/or DNA to provide broader worldwide access to HIV-1 surveillance, drug-resistance monitoring, and cure-research.

## Introduction

Worldwide, there are currently 38.4 million people living with human immunodeficiency virus type 1 (PLWH) (HIV-1), with 1.5 million new infections and approximately 650,000 AIDS-related deaths in 2021^1^. The widespread availability of assays to rapidly identify infected individuals coupled with early initiation of combination antiretroviral therapy (cART) has been a significant step in the control of HIV-1 infections with corresponding improvements in survival rates and quality of life^2^. The suppression of plasma viral loads to undetectable levels (< 20–50 copies/ml) by cART leads to improvement in immunological status characterized by the restoration of T cell numbers and effector functions^2^. Additionally, it is clinically established that HIV-1 undetectable = untransmittable^3^, meaning that PLWH who have undetectable viral loads cannot sexually transmit the virus to others. Therefore, HIV-1 treatment is a vital tool in HIV-1 prevention.

The typical first-line regimen for treatment-naïve HIV-1 + patients consists of two nucleoside reverse transcriptase inhibitors and either an integrase inhibitor, protease inhibitor, or a non-nucleoside reverse transcriptase inhibitor^3,4^. For optimal therapeutic outcome, the World Health Organization (WHO) recommends the initiation of cART immediately after diagnosis of HIV-1 infection regardless of the clinical stage and CD4 T-cell count^4^. The remarkable success of cART in supressing plasma viral load, however, does not correspond to eradication of HIV-1 infection due to the presence of latently infected long-lived cells harbouring integrated HIV-1 DNA^5,6^. This HIV-1 reservoir persists in cellular compartments including circulating peripheral blood, despite years of suppressive cART, and can fuel viral rebound upon cART interruption^6,7^. Consequently, cART is lifelong, which is very expensive and associated with adverse short- and long-term side effects.

### cART and viral load testing

The WHO recommends that patients on cART be monitored for treatment efficacy by an HIV-1 RNA plasma viral load (VL) test twice during their first year of treatment and annually thereafter^4^. VL tests help track viral suppression and identify virological failure. Effective viral load monitoring is an integral part of HIV programs in achieving UNAIDS “third 95”, a global drive which aims to correctly identify 95% of PLWH worldwide; 95% of whom should be on cART and 95% of whom, in turn, should be successfully virally suppressed by this treatment by 2030^8–10^. In addition, VL testing enables health workers to classify patients failing treatment according to the WHO recommended threshold of 1000 copies of viral RNA per ml of plasma and switch them to an alternative treatment regimen^9,11^.

**Figure 1:**
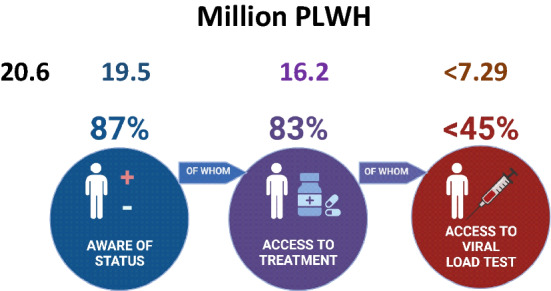
The percentages of people living with HIV + in Eastern and Southern Africa who are aware of their HIV status, who have access to combination antiretroviral therapy and who have access to viral load treatment-monitoring^1^. Created with Biorender.com.

The widespread dissemination of low-cost generic first-line HIV-1 cART, with factories that manufacture them now in operation in many LMICs means that treatment is becoming more widely available worldwide but access to high-quality assays to diagnose infection and for monitoring treatment-efficacy remains highly variable^11–14^ (Fig. 1). In 2021, only 28.7 million out of 38.4 million people living with HIV-1 (i.e. ~ 75%) worldwide, were accessing antiretroviral therapy^1^. Failure to initiate early therapy and inadequate monitoring for escape from viral suppression has resulted in continued spread of HIV-1 infection and emergence of drug-resistant strains^8^. A recent WHO survey in mostly LMICs found that 21 out of 30 countries surveyed reported HIV-1 first-line pre-treatment drug resistance levels of over 10%. These figures were at 46% on average in infants born to infected mothers, with up to 68% in these infants, in the worst-affected regions (WHO)^8^. These limitations in surveillance of populations at risk and inadequate monitoring for emergence of drug resistance will continue to hinder the achievement of the UNAIDS “third 95” goals by 2030, in LMICs, if they are not adequately addressed (Fig. 1).

### Current viral load testing platforms

Most viral load testing in LMICs is done in centralized public laboratories using expensive, government owned platforms requiring high maintenance costs, highly skilled labour, and long test turnaround times. Widely used commercial VL assays include the Real-time HIV-1 (Abbott Molecular, USA); the COBAS® AmpliPrep/COBAS® TaqMan® HIV-1 Test, v2.0 (Roche Molecular Diagnostics, Basel, Switzerland)^15^; and the Hologic Aptima® HIV-1 Quant Dx Assay on the Panther platform^15,16^. The high cost of the assays (up to $100 per test) coupled with the logistics of cold-chain transport of reagents and samples to centralized testing laboratories continues to limit the accessibility of VL testing in resource-constrained settings (RCS)^9^.

To provide more widespread coverage, the WHO has recommended expansion and decentralization of HIV treatment and care in LMICs^8,12^. Point of care (POC) assays, are being developed to allow decentralized, near-patient VL testing at medical centres, hospitals, physician offices or community health clinics at remote sites^14^. The Xpert HIV-1 Viral Load assay (Cepheid, USA), which retails around $15–20 per test, provides convenient POC automated sample processing, amplification and real time quantification in one fully integrated cartridge, and has recently received CE-IVD clearance for use in HIV-1 RNA monitoring following antiretroviral treatment. This assay provides reduced turnaround time compared to commercial laboratory-based assays to improve time to cART initiation but still requires a cold-chain system to maintain the samples and skilled personnel to process them^14^.

### Dried blood spots

Alternative sample collection methods such as dried blood spots (DBS) are increasingly being used to further VL coverage in resource constrained settings^9,11^. DBS have simplified the pre-analytical phase, and since a cold-chain system is not required to maintain their integrity, the overall cost of the VL test for diagnosis or assessment of viral resistance in research studies is significantly reduced^11^. Despite their advantages, DBS-derived viral load values do not correlate well with and underestimate the size of the plasma VL due to the use of a small volume of blood (100 µl)^9,11^. DBS-derived viral load estimates are thus not as sensitive for early identification of treatment failure^11^. Alternative sample collection systems such as plasma separation cards (PSC) and filtered dried plasma spots (FDPS), are being developed and show improved sensitivity in quantifying plasma viral load^9,11^. However, their performance in measuring the HIV-1 latent reservoir has not been assessed^11^.

### Laboratory developed assays (LDAs)

In addition to the available approved commercial assays for viral load testing, a variety of LDAs have been developed to allow simpler, less expensive testing, increased sensitivity, and earlier detection of emergence of cART resistance. The single-copy assay (SCA) has been shown to accurately detect and quantify HIV-1 RNA in plasma of suppressed individuals down to 1 copy/ml thus making it more sensitive than the available commercial assays^17,18^. Using this assay, persistent viremia is highly evident in participants with clinically undetectable plasma RNA load (generally set at < 50 copies/ml but may vary with assay) and correlates well with viral rebound following cART interruption^17,18^. The complexity of the assay, however, and requirement for ultracentrifugation greatly limits its applicability for widespread use.

Cell-associated viral load assays which test for HIV-1 RNA and DNA in Peripheral Blood Mononuclear Cells (PBMC) may also have important applications in HIV-diagnostic, surveillance, and clinical research studies (Table 1).

**Table 1:** Laboratory developed assays for monitoring of viral reservoirs.

Assay	Advantages	Limitations
Quantitative viral outgrowth assay (QVOA)^19^	Discriminates between replication-competent and defective proviruses	Expensive, labour/time-intensive, not well standardized
Viral protein spot (VIP-SPOT)^20^	Detects PBMC which express HIV Ag after stimulation	100-fold lower sensitivity than intact provirus quantification
*Alu* PCR^21^	Quantification of integrated proviral DNA	Detects only a subset of integrated proviruses
Digital droplet DNA PCR (ddDNA PCR)^22^	High sensitivity, high throughput	Possible false positive signals
Two target gag/pol RNA assay^23^	Differentiation of intact vs. defective proviruses	Complex research use assay
Next-generation sequencing (NGS) assays^24^	Proviral characterization at the single-cell level	Expensive and labour intensive
Cell-associated HIV-1 (CAH) total nucleic acid assay^25^	Earlier and more robust predictor of treatment failure and cure-therapy candidate efficacy	Generally complex sample preparation unless crude lysates or whole blood are used

In HIV-1-exposed infants, nucleic acid testing (NAT) is required to diagnose infection since passively transferred maternal antibodies preclude antibody testing^26^. However, the sensitivity of clinical NAT assays is lowered with very early cART treatment, thereby impacting early infant diagnosis^27^. DNA PCR testing is the current gold-standard for HIV diagnosis of babies using protocols for either whole blood or PBMCs^27^.

### The latent viral reservoir

An important limiting factor of current viral load assays is their inability to quantify latent reservoirs (LRs), which act as the main barrier to HIV-1 cure^7^. LRs exhibit a resting phenotype that renders them resistant to cART, are unrecognized by the immune response, and are stable over long periods^6,7^. The LR is usually established 3 to 10 days after infection and rapid initiation or intensification of cART has proved ineffective in halting the process^7^. Quantification of the LR is a crucial step in assessing the efficacy of HIV-1 cure strategies, though challenging due to variations within the HIV-1 genome^7,28^. Approaches for quantification of LR include viral outgrowth assays to measure proviral replication competence, sequencing-based assays to measure genetic intactness of HIV-1 proviruses, techniques that measure the ability of proviruses to produce viral RNA and/or proteins^24^ and cell associated (CA) viral load assays^25^ (Table 1).

### Virus culture-based assays for evaluating the latent reservoir

The quantitative viral outgrowth assay (QVOA) has long been considered the gold standard assay for quantifying intact provirus^6,7,28,29^ (Table 1). The traditional QVOA involves the activation of limiting dilutions of enriched memory CD4 + T cells using phytohemagglutinin or costimulatory antibodies such as anti-CD28 to induce the expression of HIV-1 intact proviruses^7,29^. The released viruses are then expanded by incubating the activated CD4 + T cells with PBMCs from healthy donors for approximately 3 weeks^29^ followed by quantification of p24 antigen by an Enzyme Linked Immunosorbent Assay (ELISA)^7^. Although the QVOA is highly specific and can discriminate between replication-competent and defective proviruses, the assay is expensive, labour/time-intensive, and requires a large sample volume^7,28,29^. Furthermore, the expression of the major HIV-1 coreceptor CCR5 on CD4 + T cells is highly variable among different healthy donors affecting the assay's reproducibility^29^. More recent refinements of the QVOA have included use of the SupT1- CCR5 cell line, which stably expresses CCR5^29^, the use of polarizing cytokines to improve efficiency and reliability of the assay^28^, and a switch to one-step RT-qPCR for detection of virus expression to significantly reduced the time to results^30^. Nevertheless, despite all these modifications, the high cost, long turn-around times, high variability, and random nature of virus activation which undervalues the size of the LR pool have limited the routine use of this assay in reservoir monitoring and cure studies^7^.

An alternative approach to quantifying intact proviruses or the inducible latent reservoir is the viral protein spot (VIP-SPOT) assay^20^ designed to assess what fraction of cells harbouring latent HIV-1 can be reactivated upon stimulation. The assay quantifies the frequency of CD4 + T cells that produce HIV antigen upon stimulation and their numbers were shown to be 100-fold lower than that of the corresponding intact proviruses, suggesting that most cells harbouring proviral sequences are not prone to reactivation^20^ (Table 1).

### Nucleic acid-based assays for evaluating the latent reservoir

Given the limitations of culture-based assays, PCR-based LDAs such as plasma RNA or intracellular HIV-1 RNA and DNA^7,17^ are now more frequently used for the detection and quantification of various forms of persistent HIV-1 infection^7,17,31–33^. The DNA-based assays have been shown to be an attractive proxy for the QVOA, but they may over-estimate the latent reservoir by including defective proviral sequences. The detection of low-grade viral RNA transcription also, may not necessarily correlate with full viral reproduction and may be an over-estimation of the inducible latent reservoir.

*Alu* PCR is an alternative LDA which is used for quantification of integrated proviral DNA and positively correlates with the QVOA^6,7,33^. *Alu* PCR is a high throughput 2-step nested PCR which involves the use of primers specific for HIV-1 gag and interspersed repetitive DNA elements (*Alu* sequences) in the human genome^7,21^. A major limitation of this assay is its inability to quantify a substantial proportion of the integrated provirus that exists far away from the randomly dispersed *Alu* sequences^7^ (Table 1).

Another alternative approach for sensitive detection of HIV proviral DNA is the use of droplet digital PCR (dPCR)^22^ (Table 1). With dPCR, samples are fractionated into multiple droplets and quantification of HIV-1 DNA occurs in each droplet^7^. The expression levels of the target DNA in each droplet are determined by Poisson distribution thus eliminating the complications encountered in using standard curves providing improved sensitivity and precision over qPCR^22^. The assay has recently been clinically validated across different HIV-1 subtypes^34^. Additional refinements have used multiplexed dPCR in the Intact Proviral DNA assay (IPDA), to effectively discriminate intact from defective proviruses^22,35^.

A promising approach to differentiate between full-length and truncated genomes is to target two widely spaced regions of the viral genome (Table 1). Whereas polymerase sequences are detected in both full length and truncated genomes, a significant linear correlation is observed between LTR-based HIV-1 RNA levels and PBMC-associated proviral DNA^36^. Further improvements and higher throughput in PCR and next-generation sequencing (NGS) assays have enabled proviral characterization at the single-cell level to differentiate between intact and defective proviruses, assess the host proviral integration sites, and phenotypic characterization of the host cell lineage^24,37^.

Measurement of HIV DNA in PBMCs is a valuable marker of viral reservoir size^38^. Cell-associated HIV RNA has been shown to be an important prognostic marker of disease progression in untreated patients and as an indicator of residual virus replication and the size of the dynamic viral reservoir in ART-treated patients^39^. Adult HIV-1 infections acquired under pre-exposure prophylaxis, for example, may result in low or undetectable plasma viremia and indeterminate antibody tests, for which HIV-1 DNA and cell associated RNA testing may be valuable for both diagnosis and monitoring persistence of viral reservoirs. The level of Cell Associated HIV in PBMC has also been shown to correlate with time of initiation of cART after infection and inversely correlate to time of viral rebound upon cessation of cART treatment in individuals with undetectable plasma viral loads^40^. Thus quantification of cell-associated HIV nucleic acid (CAH) will be useful for monitoring patients under therapy or those participating in HIV remission.

A recently developed CAH PCR assay has shown high sensitivity with broad cross-subtype specificity for measuring HIV-1 RNA and DNA levels in PBMCs and plasma^25^. This assay has an efficiency of > 95% and a limit of detection of approximately 3 input copies of DNA or RNA. All major HIV-1 subtypes, and a wide range of recombinants from a 127-member internationally recognized diversity panel were detected and accurately quantified. The assay is simple to use, works efficiently on crude cellular lysates and is cost-effective at approximately $4 per test.

## Discussion

Despite many years of development and evaluation in the literature, low-cost laboratory developed assays (LDAs) have not been approved internationally for HIV-1 treatment-monitoring^27,41,42^. Further applications of the approaches described above, coupled with simpler sample collection and storage matrices (e.g. dried blood spots), have considerable potential to broaden global surveillance and patient monitoring for HIV reservoirs^43^. Various programs such as the HIV Reservoir Assay Validation and Evaluation Network project^44^ (RAVEN) (https://research.vitalant.org/Investigators/Research-Interest-Articles/The-Raven-Program.aspx) and the External Quality Assurance Program Oversight Laboratory program (EQAPOL) (https://eqapol.dhvi.duke.edu/) have arisen to facilitate the validation of these assays to the same stringency as commercially-approved assays but have not yet led to international approval of LDAs.

The COVID-19 pandemic provided a blue-print for quick and effective international dissemination of PCR-based LDAs by making sequence information and protocols available for laboratories to use and adapt under emergency use authorization. SARS-CoV– 2 PCR assays were validated retrospectively and improved with time as patient reference panels became available^45^. The prospects for wider use of HIV-1 LDAs is more promising as reference reagent panels and programs are already in existence and there are a wide variety of validated or semi-validated published assays available.

## Conclusion

Much progress has been made towards meeting the WHO 95-95-95 goals to control HIV infections worldwide, but significant challenges remain, particularly in access to therapy and meaningful monitoring for the emergence of escape from viral suppression. The high cost of viral load testing, complex logistics in obtaining and reporting of timely results, limitations for access in resource-constrained settings, and inadequacy of accurate monitoring of latent reservoirs contribute to continued risk of virus spread and emergence of drug resistant strains.

Several new technologies for sensitive detection of the emergence of drug resistant strains and the measurement of latent virus reservoirs offer promising approaches to fill the existing gaps. Culture-based assays are a useful research tool for understanding parameters for virus emergence but are expensive, labor-intensive and too slow to provide significant clinical value in resource-limited settings. Some of the nucleic acid-based assays, particularly cell-based assays hold more promise, but will need to be easier to use, made more widely available at the Point-of-Care and at a lower cost. Additional support for evaluation, approval and broader distribution of these assays is needed before they can make an impact on  the control of HIV infections worldwide. The global health community should consider the many advantages of the available LDAs to address the shortfalls in HIV treatment-monitoring and cure-research access by facilitating their evaluation, approval and implementation, particularly in low and middle-income countries where the need is greatest.

## Data Availability

Detailed raw data files detailing the performance of the recently published cell-associated HIV-1 (CAH) nucleic acid PCR assay mentioned in the section on LDAs and in Table 1^25^ can be viewed via this link https://osf.io/m9yne/?view_only=47cbcfd3d39b4a89a0d758a474441e38.
